# Universal coverage in an era of privatisation: can we guarantee health for all?

**DOI:** 10.1186/1471-2458-12-S1-S1

**Published:** 2012-06-22

**Authors:** Pascale Allotey, Shajahan Yasin, Shenglan Tang, Su Lin Chong, Julius Chee Ho Cheah, Daniel D Reidpath

**Affiliations:** 1 School of Medicine and Health Sciences, Monash University, Sunway Campus Malaysia; 2 Duke Global Health Institute, Duke University, Durham, NC, USA; 3 Prince Court Medical Center, Kuala Lumpur, Malaysia

## 

A government that claims to provide universal health coverage (UHC) needs to establish that access to health services is available for the whole population for the full spectrum of services without risk of undue financial hardship. Embedded within the idea of UHC are two distinct notions. First, access to the full spectrum of health services needs to include access to preventive care through to palliative care and rehabilitative services. Second, access to services for a whole population means that everyone should be able to enjoy the benefits of the health system, regardless of individual economic, social, or geographic position.

Those in favour of UHC see health as a public good not simply an individual benefit, and they recognise that, as a consequence of this view, the implementation of UHC requires a level of regulation and a kind of investment that is inconsistent with an unconstrained free market. The challenge for government is in selecting the mix of regulatory and financing mechanisms for the chosen, universally available, health services. This also presupposes that the parcel of health services that will be available has been identified, and there are systems in place to monitor and evaluate the system.

It was around these issues that the *International Symposium on Universal Health Coverage in Malaysia*, convened by Global Public Health at the School of Medicine and Health Sciences, Monash University Sunway Campus on 3 – 4 October 2011 cohered. The symposium provided an opportunity for lively and robust discussions between the private health care sector, including private health care and insurance providers, government and academics. The proceedings of the symposium are expected to feed into the background papers for the *Second Global Symposium on Health Systems Research* to be held in Beijing, 31 October to 3 November 2012 and copies of presentations are accessible [1]. The gathering also presented the opportunity for the Monash 2011 Global Health Oration on the topic, presented by Professor Timothy Evans [2].

This supplement presents a compilation of select papers from the symposium which attempt to examine the concept of UHC from a series of different perspectives.

1. Equity and vulnerability;

2. Insurance and financing;

3. Coverage and satisfaction; and

4. Implementation.

These perspectives necessarily overlap and the insights from one perspective help to inform the considerations from another perspective.

## Equity and vulnerability

UHC is about the delivery of health care to whole populations. The long standing work on the social determinants of health identifies socio-economic stratification as one of the most significant factors affecting health and, most importantly in the context of the work in this *Supplement*, one of the most significant factors affecting access to health services: preventive, palliative, and rehabilitative. Social vulnerabilities are important because they interfere with the universality of UHC. Health systems need to be designed so that everyone can access them. It is not enough for great services to be available; if UHC is to be achieved those services must be accessible to those people usually most vulnerable to exclusion. The topic was addressed in some detail in Tim Evans’ Global Health Oration [2].

In presentations by Allotey, Ravindran, Kamarulzaman and Alvarez-Castillo, the challenges of working with vulnerable and hard to reach populations [3] were explored. The paper by Ravindran is included in this collection. Vulnerable groups are explored with examples of how social stratification negatively affects health and access to health services. In the background concept paper, Allotey et al. flip this idea of vulnerability, however, to also explore vulnerability in health systems. We know that people who are economically disadvantaged are at higher risk of poor health, and then for financial or other social reasons find it harder to access health services. In a world that expects health systems to provide universal coverage, however, one can see the issue not as one of “the poor”, “the vulnerable”, and “the disadvantaged”; rather, the vulnerability lies in the health system. Some health systems are more vulnerable to failure (ie., incapacity to deliver UHC) than others. What then are the determinants of the vulnerability of a health system? In a historical case study of South Africa, Alex van den Heever identifies a series of political choices, for instance, that made the health system more or less vulnerable to providing UHC including restrictions on risk-pooling. Seddoh and Akor also highlight similar challenges in their case study of Ghana.

Requiring a health system to provide care to a whole population carries with it significant marginal costs for the delivery of care to the most inaccessible or most at need sections of the population. The costs of delivering care to geographically remote or sparsely distributed populations can be much higher than the concentrated delivery of care in urban settings. Delivery of care to those with pre-existing, chronic conditions is often more expensive as is the treatment of rare diseases compared with common ones. This can give way to discussions of “equity versus efficiency”, or the “equity-efficiency trade off.” Reidpath et al. argue that the discussion of the trade off arises from a fundamental misunderstanding of the kind efficiency required from a health system. If UHC requires the equitable delivery of services, then the only useful measure of efficiency must include equitable delivery. A trade off may occur between equity and, say the number of people treated, if the delivery of equitable care means treating less people, but the trade off is not a trade off with efficiency.

## Insurance and financing

It is impossible to imagine a meeting about UHC that is not significantly concerned with the mechanisms for financing the delivery of health services. One of the key impediments to implementation of UHC is the lack of funds to finance the health sector coupled with inefficient allocation and use of health resources. This is exacerbated in countries with a fragmented healthcare system consisting of a public-private mix, where there are gaps in health service delivery and different levels of services (primary, secondary, tertiary) are disjointed between the public and private sector health sector. Major health policy reforms can also result in gaps in health governance which can hinder the progress towards universal coverage.

The financing mechanisms, including insurance, were explored based on regional analyses from Mahal and James and with more country specific case studies from Thailand, the Philippines and Singapore. Chua and Cheah discuss the WHO Health Financing Strategy for the Asia Pacific (2010-2015) as a framework for evaluating UHC in Malaysia using national health accounts data. They did not identify serious concerns with the financing directly, but they do observe issues around the workforce – specifically the loss of expertise from the public to the private system. And they also highlight the cost implications for UHC of a shifting burden of disease in Malaysia from disease requiring acute care with costs constrained at least by the short treatment period to chronic care of conditions such as diabetes and kidney disease. The disease transition is not peculiar to Malaysia and will have many governments worried because of the implications for health financing. Elsewhere Allotey and colleagues have argued that the increasing burden of chronic disease will require a fundamental rethink of what it means for health services to deliver care [4].

van den Heever in his historical analysis of changes in insurance and health financing regulation in South Africa observes the way that the private sector responds to government shifts. He noted positive aspects of the role of the private sector extending UHC by mobilising additional funds from those able to afford private cover, and negative aspects when the sector was permitted to entrench inequalities by excluding people with poor health from insurance cover. Because of the historical perspective van den Heever is able to track changes over time often missing in other policy analyses; something important for those interested in insurance, regulation, and financing.

Even if governments can achieve UHC, the financial challenges continue. China has seen the coverage of its population grow from 30% in 2003 to over 90% by the end of 2010 (See Tang et al.). Simultaneously there has been extraordinary increase in the cost of healthcare in China. Tang and colleagues consider a series of initiatives to curb cost escalation from changes in drug procurement mechanisms to the application of standard clinical pathways in hospitals.

## Coverage and satisfaction

Monitoring and evaluation must be integral to any successful implementation of UHC. Tang et al. describe the increase in insurance coverage of the population of China, and the simultaneous increase in healthcare costs. These kinds of effects need to be monitored. Does an increase in coverage translate into equitable use of health services across the social strata? Elsewhere Reidpath and colleagues had looked at this question with respect to urban-rural differences in health service utilisation in China [5].

Viroj and colleagues give a thorough account of the UHC scheme in Thailand that covers some 47 million people not otherwise covered by the private of government sectors. Employing data from a series of nationally representative household surveys conducted between 2003 and 2009, they were able to look at health service utilisation across wealth quintiles, answering a fundamental question about equity and access. The positive view of the Thai UHC is attributed to the quality and geographical coverage of health infrastructure, functioning financing, a functioning primary healthcare system, and zero co-payments at the point of service.

It is now accepted, however, that a health system is not about the top-down provision of care. The population will have a view about the quality, accessibility, value, and appropriateness of the services, and this needs to be taken into account. Delivery of high quality services that a population does not want is inconsistent with a modern rhetoric of health systems responsiveness. Ma and colleagues demonstrate the value of looking at residents' satisfaction with their health services. Using data from a stratified random sample of Shanghai residence they are able to identify where health systems may be under-performing, and through sub-group analyses, they can also identify whether the system is working better for some strata of the population than others. Disadvantage groups, for instance, identified lower levels of satisfaction with the health related services than less disadvantaged groups.

## Implementation

Most of the papers in this *Supplement* present insights for the implementation of UHC such as the approaches to monitoring and evaluation discussed by Tang et al., Viroj et al, and Ma et al., or the insights into financing provided by van der Heever, and Chua and Cheah. Seddoh and Akor contextualise the implementation of UHC with a case study of social insurance in Ghana. They provide critical insights into stakeholder processes and the need to engage with both politics and technical expertise.

## Concluding remarks

Universal coverage is a noble health goal especially for low and middle income countries, but it is being challenged by a range of obstacles from within and outside the health sector. From an economic viewpoint, resources are always limited in any context, universal coverage can never be open-ended and universal access to all available health services does not necessarily need to be taken as the ultimate goal. On the contrary, a systematic and dynamic approach to formulate the appropriate covered services of any universal coverage system is fundamental to its sustainability. This should ideally start with a clear, contextually specific definition of preferred health outcome goals that is used to guide the determination of health and health related services as well as service goals for achieving such outcomes, which would be key in determining a core benefit package for universal access.

Governments have a responsibility to ensure that all providers, public and private, operate appropriately and attend to patients’ needs cost effectively and efficiently. Achieving the goal of universal coverage requires expanding and improving the three facets of universality; the breadth and depth of health services, and levels of financial protection. While the primary mandate and social responsibility lies with the government, there is a growing call and recognition for the private sector to play a more substantive role. Nonetheless, there are groups of people slip through the gaps in most systems, and patterns of exclusion from services vary. In preserving and promoting health equity and equality, one has to be aware of who should benefit the most, as free public services under the umbrella of universal coverage may also be captured by the rich, who use them more than the poor, even though their need may be less.

## References

[B1] MONASH University - Panelists and Presenters | projects 2012 http://www.med.monash.edu.my/gph/projects/panelists-and-presenters.html

[B2] Global Health Oration 2011- Tim Evans.pdf 2012 http://c770304.r4.cf2.rackcdn.com/Symposium/Prof%20Tim%20-%20oration.pdf

[B3] UHC for the hard to reach_Allotey.pdf 2012 http://c770304.r4.cf2.rackcdn.com/Symposium/Prof%20Pascale.pdf

[B4] Allotey P Reidpath DD Yasin S Chan CK de-Graft A Aikins Rethinking health-care systems: a focus on chronicity Lancet 2011 377 9764 450 1 10.1016/S0140-6736(10)61856-9 21074257

[B5] Jian W Chan KY Reidpath DD Xu L China’s rural-urban care gap shrank for chronic disease patients, but inequities persist Health Aff (Millwood) 2010 29 12 2189 96 10.1377/hlthaff.2009.0989 21134919

